# Signal transducer and activator of transcription 5b: a new target of breast tumor kinase/protein tyrosine kinase 6

**DOI:** 10.1186/bcr1794

**Published:** 2007-11-12

**Authors:** Amanda M Weaver, Corinne M Silva

**Affiliations:** 1 Department of Microbiology, University of Virginia, Charlottesville, VA 22908, USA; 2 Department of Medicine, University of Virginia, Charlottesville, VA 22908, USA; 3 Cancer Center, University of Virginia, Charlottesville, VA 22908, USA

## Abstract

**Introduction:**

Signal transducers and activators of transcription (STATs) are mediators of cytokine and growth factor signaling. In recent years, STAT5b has emerged as a key regulator of tumorigenesis. STAT5b phosphorylation and activation is mediated by several kinases known to be overexpressed in breast cancer, such as epidermal growth factor receptor, HER2, and c-Src. Breast tumor kinase (Brk), also known as protein tyrosine kinase 6, is a nonreceptor tyrosine kinase expressed in more than 60% of breast cancers. Only a few substrates of the Brk tyrosine kinase have been identified, the most recent being STAT3. In the present article we investigate the potential role of Brk in the phosphorylation and activation STAT5b.

**Methods:**

To determine whether Brk can phosphorylate STAT5b, transient transfection and *in vitro *kinase assays were performed. Luciferase reporter assays were used to measure Brk-induced STAT5b transcriptional activity. siRNA technology was utilized to investigate the biological significance of Brk-induced activation of STAT5b in breast cancer cell models.

**Results:**

Phosphospecific antibodies, mutational analysis, and *in vitro *kinase assays demonstrated that Brk specifically mediated STAT5b phosphorylation at the activating tyrosine, Y699. Transient transfection of Brk into the Brk-negative BT-549 breast cancer cell line enhanced STAT5b transcriptional activity, as measured by a STAT5-specific luciferase reporter. Furthermore, overexpression of kinase active c-Src enhanced Brk-induced STAT5b transcriptional activity. In Brk-positive breast cancer cell lines BT-20 and SKBr3, knockdown of Brk protein or of STAT5b protein using siRNA methodology resulted in a decrease in DNA synthesis. Knockdown of Brk and STAT5b together did not further decrease DNA synthesis compared with each alone, suggesting that Brk and STAT5b converge on the same pathway, ultimately leading to cellular proliferation.

**Conclusion:**

Our studies demonstrate that Brk phosphorylates STAT5b on Y699, leading to increased STAT5b transcriptional activity. Furthermore, analysis of DNA synthesis suggests that STAT5b and Brk are converging upon the same proproliferative signaling pathway in breast cancer cells. We propose that Brk, like other tyrosine kinases, signals downstream to STAT5b to mediate proliferation of breast cancer cells. These results further establish STAT5b as well as Brk as potential targets for breast cancer therapy.

## Introduction

As mediators of cytokine-induced and growth factor-induced gene expression, signal transducers and activators of transcription (STATs) are involved in cellular differentiation, proliferation, and survival. Upon cytokine or growth factor binding to its receptor, the latent cytoplasmic STAT proteins are recruited to the receptor complex resulting in STAT activation by either receptor tyrosine kinases or nonreceptor tyrosine kinases such as Janus kinases or c-Src. Activation of STAT proteins requires phosphorylation on a conserved tyrosine residue located in the carboxy terminus. Phosphorylation of this tyrosine leads to phosphotyrosine–Src homology domain 2-mediated reciprocal dimerization. The activated STAT dimer then translocates to the nucleus and binds to a STAT consensus DNA element, resulting in gene transcription. The STAT family consists of seven members that can be divided into two categories: those that respond to cytokine signals (STAT2, STAT4, STAT6), and those that respond to cytokine and growth factor signals (STAT1, STAT3, STAT5a, STAT5b) [1-3].

Although STAT5a and STAT5b play a fundamental role in normal growth and development of the mammary gland, both proteins are overexpressed or constitutively activated in cancers, including some breast cancer tumors [4-9]. Owing to their ability to regulate the expression of genes involved in cell-cycle regulation (cyclin D_1_, c-*myc*, and p21) and cellular survival (Bcl-X_L_), STAT5a and STAT5b have emerged as possible targets for cancer therapeutics [10]. Recent evidence indicates that STAT5b, but not STAT5a, has a proproliferative role in breast cancer, head and neck cancer, and prostate cancer [11-14]. Since STAT5b mediates breast cancer proliferation, identification of kinases that increase STAT5b activity is critical to identifying potential therapeutic targets.

Breast tumor kinase (Brk) is a nonreceptor tyrosine kinase originally isolated from an involved axillary node of a patient with metastatic breast cancer, and is expressed in more than 60% of breast cancers [15,16]. With 46% amino acid identity to c-Src, Brk is distantly related to the Src family of tyrosine kinases [17,18]. Although normally expressed in the gastrointestinal tract, expression of Brk is not detected in the normal mammary gland [16,19]. Stable transfection of Brk in the immortalized nontransformed human mammary cell lines HB4a and MCF10A, however, leads to sensitization to epidermal growth factor and results in a partially transformed phenotype [20]. Brk also enhances epidermal growth factor-induced ErbB3 and Akt phosphorylation in the HB4a cells [21]. Furthermore, knockdown of the Brk protein decreases proliferation of breast cancer cell lines [22]. Given its role in breast cancer cell proliferation, survival, and tumorigenesis, identification of the substrates of this tyrosine kinase is of utmost importance.

Although STAT5b is involved in cancer proliferation, mutations of STAT5b to account for this increased biological activity have not been identified. Alternatively, increased STAT5b activation results from the overexpression and/or the constitutive activation of tyrosine kinases, such as the epidermal growth factor receptor (EGFR), c-Src, and the fusion protein Bcr/Abl [4,6]. Since all identified Brk substrates are also substrates for c-Src, we examined the ability of Brk to mediate STAT5b phosphorylation, a known c-Src substrate.

In the studies presented here, the ability of Brk to phosphorylate and activate STAT5b and the biological significance of this activation were investigated. Exogenous expression of Brk and STAT5b demonstrated that Brk mediates the phosphorylation of the activating tyrosine, Y699. Furthermore, an *in vitro *kinase assay determined that Brk can directly phosphorylate STAT5b on Y699. Subsequently, this Brk-mediated STAT5b phosphorylation leads to STAT5b transcriptional activity, and this activity is further increased by kinase active c-Src. The results of siRNA experiments suggest that Brk and STAT5b are in the same signaling pathway, which ultimately leads to the proliferation of breast cancer cells. These studies further support targeting STAT5b as a potential breast cancer therapeutic.

## Materials and methods

### Cell lines and transient transfections

The human breast cancer cell lines SKBr3, BT-20, BT-549, MDA-MB-468, and T47D were obtained from the American Type Culture Collection (Manassas, VA, USA). Cells were maintained in DMEM plus 10% FCS and were passaged twice per week. Mouse embryo fibroblasts (MEF5^-/-^) from STAT5a/b knockout mice (provided by Dr J Ihle, St Jude Children's Hospital, Memphis, TN, USA) were passaged twice per week and maintained in DMEM plus 10% FCS. Cells were transfected with STAT constructs [23], Brk constructs (generous gift from Dr C Lange, University of Minnesota, Minneapolis, MN, USA), and c-Src constructs as previously described [24], using LipofectAMINE and PLUS reagent according to the manufacturer's instructions (Invitrogen, Gaithersburg, MD, USA).

### Reagents

The polyclonal STAT5a-specific and STAT5b-specific antibodies were developed in our laboratory, as previously described [23]. Polyclonal anti-STAT3 antibodies, polyclonal anti-Brk antibodies, and monoclonal antiphosphotyrosine antibodies (PY-99) were obtained from Santa Cruz Biotechnology (Santa Cruz, CA, USA). The monoclonal anti-β-actin antibody was from Sigma (St Louis, MO, USA). The antiphospho-STAT5a/b (Y694/Y699) antibody was developed in conjunction with Aves Labs (Tigand, OR, USA) as described elsewhere (EM Fox, T Bernaciak, J Wen, A Weaver, M Shupnik, CM Silva - unpublished data). The protease inhibitor cocktail was from Calbiochem (San Diego, CA, USA). The acrylamide was obtained from Bio-Rad (Hercules, CA, USA), and the prestained molecular weight marker was from Invitrogen. Except as noted, other reagents were of either reagent grade or molecular biological grade from Sigma.

### Immunoprecipitations and immunoblotting

Cells were lysed in 150 mM NaCl, 5 mM ethylenediamine tetraacetic acid, 1% Triton X-100, 1% deoxycholate, 50 mM Tris (pH 7.4), containing protease inhibitors and phosphatase inhibitors. Lysates were incubated with the indicated antibody overnight at 4°C, and protein A-agarose (Santa Cruz Biotechnology) was added for an additional 1 hour at 4°C. Agarose pellets were washed three times in detergent lysis buffer, and the bound proteins were removed by boiling in 1 × Laemmli buffer. Proteins were separated on polyacrylamide gels and were analyzed as previously described [12].

### Luciferase assay

MEF5^-/- ^and BT-549 cells were transfected with the Spi2.1-containing luciferase reporter plasmid. Forty-eight hours post transfection, lysates were prepared and luciferase activity was measured [24]. The luciferase values (arbitrary units), as measured by a Berthold Luminometer (Berthold, Oak Ridge, TN, USA), were normalized to total protein.

### Recombinant protein

STAT5b or Y699F cDNA was cloned into the pGEX4T-1 plasmid (Amersham, Piscataway, NJ, USA) using the *Eco*RI and *Not*I restriction sites. The GST, GST-STAT5b, and GST-Y699F plasmids were generated and transformed into *Escherichia coli *BL21. Protein expression was induced by 1 mM isopropyl-beta-D-thiogalactopyranoside (IPTG in Luria broth (LB) broth at 18°C for 18 hours. Bacterial cells were lysed in 1 × PBS containing 1 mM ethylenediamine tetraacetic acid, 5 mM dithiothreitol, 1.5% sarkosyl, 1% Triton, 2 mM phenylmethylsulfonyl fluoride (PMSF), and 1 μg/ml pepstatin followed by sonication. GST, GST-wtSTAT5b, and GST-Y699F were isolated using glutathione agarose beads (Sigma) following the manufacturer's instructions. Following elution with excess glutathione (50 mM Tris–HCl/10 mM glutathione, pH 8.0), the recombinant proteins were dialyzed in 1 × Tris-buffered saline/10% glycerol. Protein concentration was determined by the Bio-Rad Protein Assay (Bio-Rad, Hercules, CA, USA).

### *In vitro *kinase assay

Purified recombinant Brk (Upstate, Billerica, MA, USA) and purified recombinant GST, GST-STAT5b, or GST-Y699F were incubated with 20 nM ATP in reaction buffer (100 mM Tris–HCl, pH 7.4, 125 mM MgCl_2_, 25 mM MnCl_2_, 2 mM ethylene glycol tetraacetic acid (EGTA), 0.25 mM NaVO_4_, 2 mM dithiothreitol) at 30°C for 30 minutes. An equal volume of 2 × Laemmli was added to end the reaction. Phosphorylation of STAT5b was analyzed by immunoblotting with our specific phospho-Y694/Y699 STAT5a/b antibody.

### RNA isolation and reverse transcriptase polymerase chain reaction

Total RNA was isolated from the breast cancer cell lines using the RNeasy mini kit (Qiagen, Valencia, CA, USA) following the manufacturer's instructions. The cDNA was generated using iScript cDNA synthesis (Bio-Rad). The cDNA was amplified using primers for Brk or β-actin as described by Kasprzycka and colleagues [25].

### Small interfering RNA methodology

Knockdown of Brk and/or STAT5b was performed using the siGenome SMARTpool duplex (Dharmacon, Lafayette, CO, USA) transfected with Oligofectamine (Invitrogen) according to the manufacturer's instructions.

### DNA synthesis assay

Following 48 hours of transfection with siRNA, SKBr3 cells or BT-20 cells were serum starved for an additional 18 hours and were then incubated with 100 μM bromodeoxyuridine (BrdU) for 6 hours. Cells were fixed and permeabilized as previously described [12]. Cells were blocked in 20% goat serum/PBS for 20 minutes at 37°C, and then were incubated with anti-BrdU-Alexa-Fluor 594 (Molecular Probes, Carlsbad, CA, USA) for 1 hour at 37°C. BrdU incorporation was visualized using a Leica DM RBE Fluorescence microscope (model RS232C; Leica Microsystems, Bannockburn, IL, USA).

## Results

### STAT tyrosine phosphorylation mediated by breast tumor kinase

**Figure 1:**
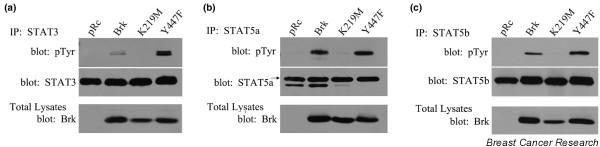
Breast tumor kinase mediates tyrosine phosphorylation of signal transducers and activators of transcription. Breast tumor kinase (Brk) mediates tyrosine phosphorylation of signal transducer and activator of transcription (STAT)3, STAT5a, and STAT5b. STAT5a/b knockout mouse embryo fibroblast (MEF5^-/-^) cells were transfected with **(a) **STAT3, **(b) **STAT5a, or **(c) **STAT5b along with either pRc (vector), Brk, K219M Brk (kinase inactive), or Y477F Brk (constitutively active). Immunoprecipitations (IPs) for STAT3, STAT5a, or STAT5b were performed using antibodies specifically directed against each STAT and were analyzed by immunoblotting with antibodies directed toward STAT3, STAT5a, STAT5b (middle), or phosphotyrosine (top). Total lysates were analyzed for the expression of the Brk construct by immunoblotting with anti-Brk antibody (bottom).

Previous studies have demonstrated that STAT5a and STAT5b are tyrosine phosphorylated by the EGFR and c-Src kinases, two kinases that are involved in breast cancer [12,26,27]. Given the recent evidence that Brk phosphorylates STAT3 [1], we investigated whether this kinase could also mediate the phosphorylation of the related STAT5a and STAT5b proteins. Mouse embryo fibroblasts from STAT5a/b knockout mice (MEF5^-/-^) were transfected with STAT3, STAT5a, or STAT5b along with various Brk expression vectors. MEF5^-/- ^cells were chosen because these cells do not express endogenous Brk (Figure 1), STAT5a, or STAT5b. Immunoprecipitations were performed for the transfected STAT using specific antibodies and were analyzed for tyrosine phosphorylation by immunoblotting. Additionally, total lysates were analyzed for the expression of the Brk constructs.

In agreement with previous findings, Figure 1a demonstrates that wildtype Brk and constitutively active Brk (Y447F) were able to mediate STAT3 tyrosine phosphorylation, while the kinase inactive Brk (K219M) or vector (pRc) demonstrated no detectable tyrosine phosphorylation [1]. Importantly, Brk and the Y447F Brk, but not the K219M Brk, were also able to mediate STAT5a and STAT5b tyrosine phosphorylation (Figure 1b,c). These data demonstrate for the first time that Brk mediates tyrosine phosphorylation of STAT5a and STAT5b in addition to STAT3. Since our previous studies demonstrated that STAT5b, but not STAT5a, elicits a proproliferative effect in breast cancer cells [12], and other studies have shown that Brk also increases proliferation of breast cancer cells [22], we focused our efforts on Brk-mediated activation of STAT5b.

### Phosphorylation of STAT5b by breast tumor kinase

While phosphorylation of Y699 is required for transcriptional activity, three tyrosines located in the transactivation domain can also be phosphorylated and modulate STAT5b activity [23]. These tyrosines are either positive (Y725) or negative (Y740 and Y743) regulators of STAT5b activity, and their phosphorylations are mediated by c-Src (Y699 and Y725) and the EGFR (Y699, Y725, Y740, and Y743) [12,27]. We investigated the ability of Brk to phosphorylate each of the four identified tyrosine sites, starting with the required activating tyrosine, Y699. MEF5^-/- ^cells were transfected with STAT5b along with Brk constructs, and STAT5b immunoprecipitations were analyzed using the antiphospho-Y699 STAT5b-specific antibody.

**Figure 2:**
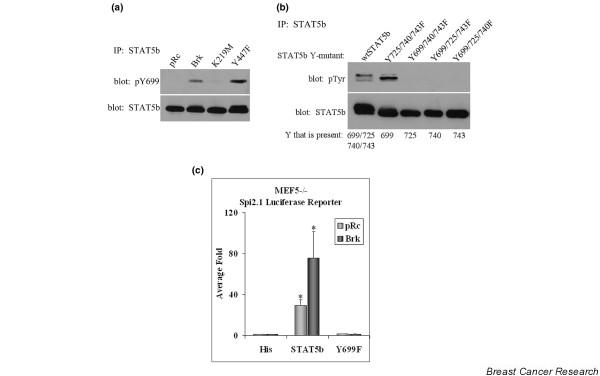
Signal transducer and activator of transcription 5b tyrosine phosphorylation and transcriptional activation. **(a) **Mouse embryo fibroblast (MEF5^-/-^) cells were transfected with signal transducer and activator of transcription 5b (STAT5b) along with pRc (vector), breast tumor kinase (Brk), K219M, or Y447F. Immunoprecipitations (IPs) were performed using antibodies specifically against STAT5b and were analyzed by immunoblotting with antibodies directed toward phospho-Y699 STAT5b (top) or STAT5b (bottom). **(b) **MEF5^-/- ^cells were transfected with various STAT5b constructs along with constitutively active Y447F Brk. Immunoprecipitations (IPs) were performed as above and were analyzed via immunoblotting with antiphosphotyrosine antibody (top) or anti-STAT5b antibody (bottom). **(c) **MEF5^-/- ^were transfected with the STAT5-specific Spi2.1 promoter luciferase construct and either His-vector, His-STAT5b, or His-Y699F with or without Brk. Luciferase activity was measured and normalized to total protein. Values from four independent experiments performed in triplicate reported as the fold induction over His, pRc ± standard error: His, pRc (1.00 ± 0.00) and Brk (1.05 ± 0.39); STAT5b, pRc (28.89 ± 5.51) and Brk (75.48 ± 25.79); Y699F, pRc (1.17 ± 0.14) and Brk (0.93 ± 0.27). Student's *t *test was used to determine statistical significance between STAT5b pRc and STAT5b Brk. *P = 0.0123.

Both Brk and Y447F Brk were able to mediate the phosphorylation of Y699, while it was not detectable in vector cells or K219M Brk-transfected cells (Figure 2a). To determine whether Brk mediates the phosphorylation of tyrosines other than Y699, MEF5^-/- ^cells were transfected with Brk along with STAT5b mutants in which all but one of the four tyrosines was mutated. Brk induced detectable tyrosine phosphorylation of wildtype STAT5b and the Y725/740/743F mutant (with intact Y699), confirming the results with the antiphospho-Y699-specific antibody. None of the other STAT5b mutant constructs, however, were phosphorylated by Brk (Figure 2b). These results demonstrate that Brk primarily mediates Y699 phosphorylation on STAT5b and not the three transactivation domain tyrosines.

### Breast tumor kinase mediates STAT5b transcriptional activity

Since Y699 phosphorylation is required for the transcriptional function of STAT5b, we analyzed the ability of Brk to mediate STAT5b transcriptional activity. MEF5^-/- ^cells were transfected with the STAT5-specific luciferase reporter plasmid (Spi2.1-luc) along with His-vector, STAT5b, or Y699F plus or minus Brk. Since the MEF5^-/- ^cells express endogenous STAT1 and STAT3, the specificity of this reporter was demonstrated by the inability of the His-vector to mediate luciferase activity (Figure 2c). The expression of STAT5b increased Spi2.1-mediated luciferase activity, and the cotransfection of Brk significantly increased this transcriptional activity further. As seen previously, the Y699F STAT5b mutant was unable to mediate STAT5b transcriptional activity, and the addition of Brk had no effect. These results demonstrate not only that Brk mediates STAT5b transcriptional activity, but that Y699 is required.

### Breast tumor kinase directly phosphorylates Y699 of STAT5b *in vitro*

**Figure 3:**
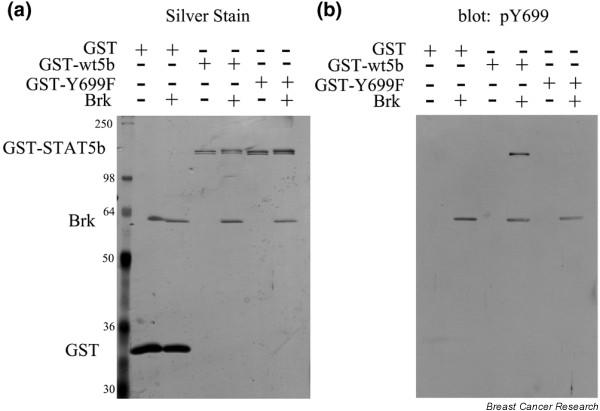
***In vitro *Y699 phosphorylation by breast tumor kinase**. *In vitro *Y699 phosphorylation of signal transducer and activator of transcription 5b (STAT5b) by breast tumor kinase (Brk). Purified recombinant GST, GST-STAT5b, or GST-Y699F were incubated with (+) or without (-) purified recombinant Brk in reaction buffer containing 20 nM ATP at 30°C for 30 minutes. An equal volume of 2 × Laemmli buffer was added to end the reaction and the samples were run on SDS-PAGE. Gels were analyzed via **(a) **silver stain and **(b) **by immunoblotting with an antiphospho-Y699 STAT5b-specific antibody.

To determine whether Brk could mediate the phosphorylation and activation of STAT5b directly, or whether other tyrosine kinases known to activate STAT5b (such as EGFR and c-Src) were required, an *in vitro *kinase assay was performed. Purified recombinant Brk was incubated with purified recombinant GST, GST-STAT5b, or GST-Y699F. The *in vitro *kinase reaction was analyzed via silver stain (Figure 3a) and by immunoblotting with the antiphospho-Y699 STAT5b-specific antibody (Figure 3b). The silver stain illustrates that GST, GST-STAT5b, GST-Y699F, and Brk were all expressed (Figure 3a). Furthermore, the specificity of the phospho-Y699 STAT5b antibody was confirmed by its inability to recognize the recombinant GST-Y699F protein (Figure 3b). Phospho-Y699 STAT5b antibody, however, detected phosphorylated Brk in this assay. Immunoblotting with this antibody demonstrated that the GST-STAT5b was phosphorylated on Y699 in the presence of, but not in the absence of, Brk. This result demonstrates that Brk is able to directly phosphorylate Y699 on STAT5b *in vitro*.

### Breast tumor kinase expression in human breast cancer cell lines

**Figure 4:**
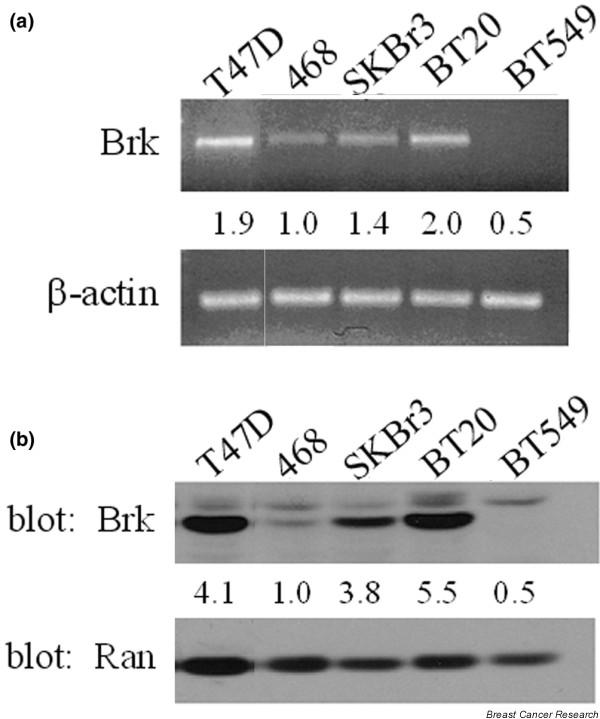
Breast tumor kinase expression in breast cancer cell lines. **(a) **Total RNA was isolated from the breast cancer cell lines using the RNeasy kit (Qiagen), and the cDNA was generated using iScript cDNA synthesis (Bio-Rad). The cDNA was amplified using primers specifically for breast tumor kinase (Brk) or β-actin. The relative level of Brk mRNA was determined using densitometric analysis and was normalized to the amount of β-actin. The level of Brk mRNA in the MDA-MB-468 cell line was set to 1; numbers indicate the fold increase compared with the 468 cells. **(b) **Whole-cell lysates were prepared from the breast cancer cell lines. Lysates were immunoblotted with anti-Brk antibody (top) and with anti-Ran antibody (bottom). The relative expression of Brk protein was determined using densitometric analysis and was normalized to the amount of Ran. The expression of Brk protein in the MDA-MB-468 cell line was set to 1; numbers indicate the fold increase compared with the 468 cells.

While studies in the MEF5^-/- ^cells allowed us to characterize the role of Brk in STAT5b activation, we continued our studies in breast cancer cell lines as a means to investigate the biological relevance of Brk-mediated STAT5b activation. We determined the relative expression of Brk by analyzing mRNA and protein levels in a panel of frequently studied breast cancer cell lines. The total mRNA was isolated from each cell line, cDNA was generated, and primers specific for either Brk or β-actin were used for amplification [25]. In each sample, the relative expression of Brk mRNA was determined by normalizing to the amount of β-actin. The expression of Brk mRNA in the MDA-MB-468 cell line was set to 1 (Figure 4a) because this cell line expressed the lowest detectable amount of Brk mRNA. While BT-20 cells and T47D cells had the highest mRNA levels, SKBr3 cells and MDA-MB-468 cells expressed moderate levels, and BT-549 cells had no detectable Brk mRNA.

The amount of Brk protein in detergent cell lysates from each cell line was determined by immunoblotting. The relative expression of Brk protein was determined by normalizing to the nuclear import protein Ran, shown previously as a valid normalization control in breast cancer cell lines [28]. Again the amount of Brk protein in the MDA-MB-468 cell line was set to 1 (Figure 4b) because this cell line had the lowest detectable amount of Brk protein. Brk was highly expressed in BT-20 cells and T47D cells, was moderately expressed in SKBr3 cells and MDA-MB-468 cells, and was not detectable in BT-549 cells. Relatively high levels of Brk expression were seen in both the estrogen-receptor-positive T47D cells and the estrogen-receptor-negative BT-20 cells. Furthermore, while MDA-MB-468 cells, BT-20 cells, and BT-549 cells all express the EGFR, they expressed different amounts of Brk.

### Breast tumor kinase mediates STAT5b activity in breast cancer cells

**Figure 5:**
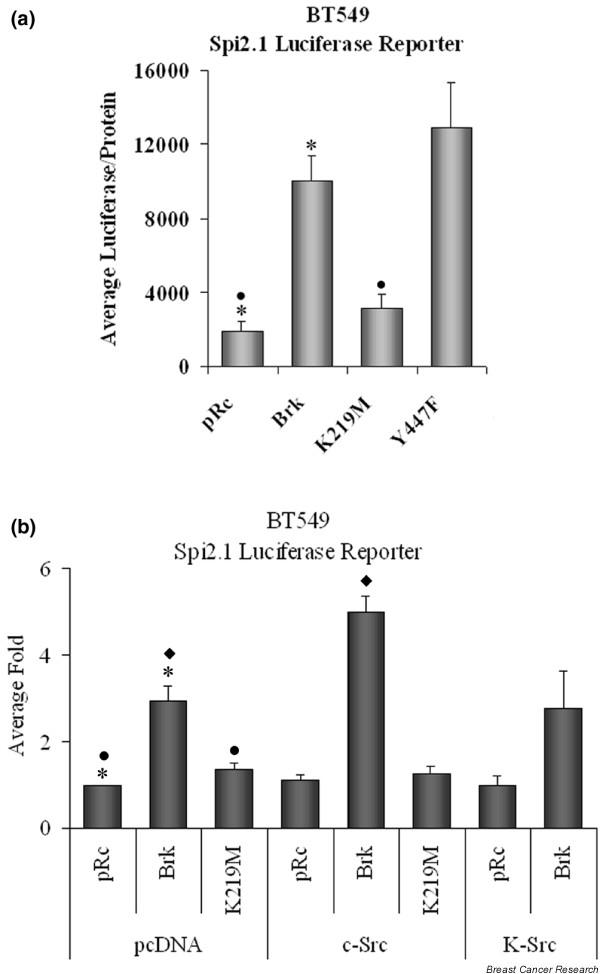
Breast tumor kinase mediates endogenous signal transducer and activator of transcription 5b transcriptional activity. **(a) **BT-549 cells were transfected with the Spi2.1 promoter luciferase construct along with pRc, breast tumor kinase (Brk), K219M, or Y447F expression vectors. Luciferase activity was measured and normalized to total protein. Values from four independent experiments carried out in triplicate were reported as average luciferase/protein ± standard error: pRc (1,859.37 ± 592.24); Brk (10,031.94 ± 1,359.68); K219M (3,109.82 ± 777.57), Y477F (12,878.57 ± 2,464.26). Student's *t *test was used to determine statistical significance between pRc and Brk, and between pRc and K219M. *P < 0.0001, • *P *= 0.0430. **(b) **BT-549 cells were transfected with the Spi2.1 promoter luciferase construct along with pRc, Brk, or K219M, and either pcDNA, c-Src, or K-Src expression vectors. Luciferase activity was measured and normalized to total protein. Values from four independent experiments carried out in triplicate were reported as the average fold induction over pcDNA, pRc ± SE: pcDNA, pRc (1.00 ± 0.00), Brk (2.92 ± 0.39), and K219M (1.36 ± 0.13); c-Src, pRc (1.10 ± 0.12), Brk (5.01 ± 0.38), and K219M (1.24 ± 0.18); K-Src, pRc (0.99 ± 0.19) and Brk (2.76 ± 0.89). Student's *t *test was used to determine statistical significance between pcDNA pRc and pcDNA Brk, between pcDNA pRc and pcDNA K219M, and between pRc Brk and c-Src Brk. *P = 0.0027, • *P *= 0.0322, ◆ *P *< 0.0086.

To examine the ability of Brk to mediate the transcriptional activity of endogenous STAT5b in a breast cancer cell line, the BT-549 cells, which have no detectable endogenous Brk, were utilized. The STAT5-specific Spi2.1 luciferase reporter with or without Brk was transfected into the BT-549 cells. While there was a detectable basal level of transcriptional activity, Brk significantly increased endogenous STAT5b transcriptional activity, as did the constitutively active Brk (Y447F) (Figure 5a). Although not nearly as effective, the kinase inactive Brk (K219M) did significantly increase the STAT5b luciferase activity compared with vector alone. This result is not necessarily unexpected since a role of the kinase inactive Brk in other cell models has been reported (see Discussion).

Since Brk and c-Src are both able to phosphorylate STAT5b on Y699, the potential cooperation between Brk and c-Src was investigated. As previously, Brk dramatically enhanced STAT5b transcriptional activity, and the K219M Brk was also able to significantly enhance this activity (Figure 5b). Unexpectedly, exogenous overexpression of c-Src did not enhance STAT5b transcriptional activity (Figure 5b). c-Src overexpression enhanced Brk-induced STAT5b transcriptional activity, however, indicating that Brk and c-Src together can enhance STAT5b transcriptional activity. The kinase activity of Brk was necessary for this collaboration as the addition of c-Src to K219M Brk did not enhance STAT5b activity. Likewise, the kinase inactive c-Src (K-Src) failed to significantly enhance Brk-induced STAT5b transcriptional activity, suggesting that the kinase activity of c-Src is also necessary for this cooperation between Brk and c-Src. While Brk and c-Src individually have different effects on STAT5b transcriptional activity, these two tyrosine kinases together increase STAT5b transcriptional activity. These data suggest that Brk and c-Src do not merely substitute for one another in activating STAT5b, but rather that Brk can function independently as well as together with c-Src.

### STAT5b and breast tumor kinase involvement in DNA synthesis

To determine the biological significance of the Brk-mediated increase in STAT5b activity, the role of Brk and STAT5b, individually and together, on DNA synthesis in breast cancer cell lines was investigated. Since Brk signals downstream of the EGFR [20,21,30,30] and HER2 [29] tyrosine kinases, the EGFR/HER2-overexpressing SKBr3 breast cancer cell line and the EGFR-overexpressing BT-20 breast cancer cell line were chosen for these studies. Both cell lines express the c-Src and Brk tyrosine kinases as well as STAT5b. To investigate the effect on proliferation, siRNA technology was used to knockdown Brk or STAT5b protein levels, individually or together, and then the effect on DNA synthesis was measured by BrdU incorporation. Lysates were analyzed via immunoblotting to determine significant and specific knockdown of Brk and STAT5b.

**Figure 6:**
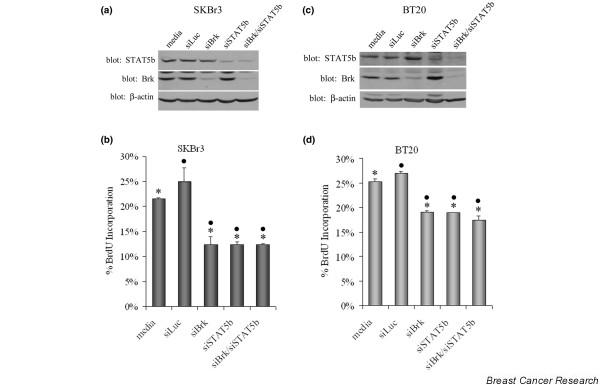
**Breast tumor kinase and signal transducer and activator of transcription 5b roles in DNA synthesis**. Knockdown of breast tumor kinase (Brk) and/or signal transducer and activator of transcription 5b (STAT5b) in SKBr3 cells **(a, b) **or BT-20 cells **(c, d) **was performed using the Dharmacon siGenome SMARTpool duplex for each protein as per the manufacturer's instructions. (a, c) Seventy-two hours after transfection of the siRNA, cells were lysed. Total lysates were analyzed via immunoblotting with anti-STAT5b (top), anti-Brk (middle), or anti-β-actin (bottom) antibodies. (b, d) Sixty-six hours after transfection of the siRNA, bromodeoxyuridine (BrdU) was added to the medium for 6 hours. The cells were fixed and stained with a fluorescent antibody against BrdU. Cells were scored for BrdU incorporation and graphed. Between 160 and 200 cells were counted for each treatment group. (b) Average percentage of BrdU incorporation ± standard error from three independent experiments performed in triplicate for the SKBr3 cells: media (21.54 ± 0.16), siLuc (24.98 ± 2.78), siBrk (12.37 ± 1.65), siSTAT5b (12.37 ± 0.51), siBrk/siSTAT5b (12.39 ± 0.21). Student's *t *test was utilized to determine statistical significance between media and siBrk, siSTAT5b, or siBrk/siSTAT5b, and between siLuc and siBrk, siSTAT5b, or siBrk/siSTAT5b. *P ≤ 0.0050, • *P *< 0.0025. (d) Average percentage of BrdU incorporation ± standard error from three independent experiments performed in triplicate for the BT-20 cells: media (25.28 ± 0.55), siLuc (26.87 ± 0.52), siBrk (18.97 ± 0.32), siSTAT5b (18.87 ± 0.03), siBrk/siSTAT5b (17.37 ± 0.88). Student's *t *test was utilized to determine statistical significance between media and siBrk, siSTAT5b, or siBrk/siSTAT5b, and between siLuc and siBrk, siSTAT5b, or siBrk/siSTAT5b. *P ≤ 0.0016, • *P *< 0.0007.

As shown in Figure 6a,c, knockdown of Brk did not effect the expression of STAT5b and knockdown of STAT5b did not effect the expression of Brk. Furthermore, knockdown of Brk and/or STAT5b did not effect the expression of HER2, EGFR, c-Src, STAT3, or STAT1 (data not shown). Knockdown of Brk (siBrk) inhibited DNA synthesis in both SKBr3 cells and BT-20 cells by 47% and 31%, respectively (Figure 6b,d). Knockdown of STAT5b (siSTAT5b) significantly decreased DNA synthesis of both cell lines to an extent similar to that seen with Brk knockdown. Importantly, the knockdown of Brk and STAT5b together did not further decrease DNA synthesis compared with either single knockdown, suggesting that signaling mediated by Brk and STAT5b are contributing to the same proproliferative pathway.

## Discussion

Although traditionally known for its role in growth hormone signaling and mammary gland development, STAT5b has emerged as a therapeutic target due to its pivotal role in cancer. Inhibition of STAT5b, but not of STAT5a, in xenograft models of head and neck carcinomas via antisense oligonucleotides repressed tumor growth and hindered expression of the STAT5-regulated genes cyclin D_1 _and Bcl-X_L _[13,14]. STAT5b is more abundantly expressed than STAT5a in prostate cancer and breast cancer cell lines [12,31]. Inhibition of STAT5b via dominant-negative constructs and siRNA technology decreases DNA synthesis (Figure 6) [32], while exogenous expression of a basally active STAT5b mutant (Y740/743F) increases DNA synthesis of breast cancer cells [12]. Together, these data establish a fundamental role for STAT5b in the process of breast cancer tumorigenesis.

Since activating mutations in STAT5b have not been found in breast cancer, it is important to look upstream to identify the kinases that regulate STAT5b, thus potentially leading to its increased activity in breast cancer cells. Both STAT5b and STAT3 are activated by several kinases overexpressed in breast cancer, including the EGFR, HER2, and c-Src. STAT3 was recently shown to also be phosphorylated by the nonreceptor tyrosine kinase Brk [1]. Since Brk is expressed in more than 60% of breast tumors but not in normal mammary tissue, it has been suggested to be a potential therapeutic target for breast cancer. Since evidence supports the involvement of both Brk and STAT5b in breast cancer proliferation, we investigated the ability of Brk to phosphorylate STAT5b and the biological significance of this activation.

Using synthetic peptides containing consensus motifs for the EGFR, insulin receptor, c-Src, or Abl tyrosine kinases, it was determined that the c-Src-preferred synthetic peptide is the best Brk substrate [17]. Given these data, it is not surprising that all of the identified Brk substrates (Sam68, SLM-1, SLM-2, STAP-2, paxillin, STAT3) are also c-Src substrates [1,30,33-35]. We have identified two more Brk substrates, STAT5a and STAT5b, which are also c-Src substrates (Figure 1). c-Src can mediate the phosphorylation of Y699 and Y725, however, Brk mediates Y699 phosphorylation but not Y725 phosphorylation (Figure 2b) [12,27]. A previous study demonstrated that Brk mediated phosphorylation of STAT3, but not of STAT1, STAT2, STAT5, or STAT6 [1]. These studies were performed in COS1 cells, however, not in breast cancer cells, and *in vitro *kinase assays were not performed for STATs other than STAT3. Using our previously characterized STAT5b-specific antibodies, we demonstrated here that Brk can also directly phosphorylate Y699 on STAT5b in breast cancer cell lines and in an *in vitro *kinase assay.

Exogenous expression of Brk in the Brk-negative breast cancer cell line BT-549 increased endogenous STAT5b transcriptional activity. Interestingly, the catalytically inactive K219M Brk mutant also significantly increased STAT5b transcriptional activity compared with vector alone, although not to the extent seen with wildtype Brk or the constitutively active (Y447F). In fact, Harvey and Crompton have previously reported that the kinase-inactive Brk mutant (K219M) could increase the proliferation of T47D cells compared with vector [22]. Since the K219M mutation disrupts the ATP-binding motif, but not the Src homology domain 2 or the Src homology domain 3, these data suggest that Brk has a role in intracellular signaling that does not require its kinase activity. In these cases, Brk may function as an adaptor protein. Finally, although the constitutively active Y447F Brk mutant was able to increase STAT5b transcriptional activity, it was not significantly higher than that seen with wildtype Brk. This mutation is at the presumptive autoinhibitory tyrosine phosphorylation site of Brk (Y447), equivalent to that identified in c-Src (Y527). Although the Y447F Brk increases phosphorylation of a synthetic peptide, the autophosphorylation of the activating tyrosine in Brk (Y342) is comparable with that seen with wildtype Brk [17,36]. Nevertheless, the Y447F Brk mutant has decreased transforming potential when compared with wildtype Brk in NIH3T3 cells [20]. Together, these data as well as the results we have presented here suggest that the regulation of Brk is more complex than originally thought, and probably involves its role as a kinase and an adaptor protein depending on the cell context.

Since both c-Src and Brk tyrosine kinases are frequently overexpressed in breast cancer and since they both mediate Y699 phosphorylation of STAT5b, there is potential for these kinases to either substitute for one another or work together to activate STAT5b. As shown in Figure 5b, exogenous overexpression of c-Src, unlike Brk, did not enhance STAT5b transcriptional activity in the BT-549 cells, although we have reported that it does in other cell lines [12]. Exogenous expression of c-Src along with Brk, however, enhanced STAT5b transcriptional activity to a level greater than that with Brk alone. As the kinase-inactive c-Src did not enhance Brk-mediated STAT5b transcriptional activity, c-Src kinase activity may play a role in increasing the phosphorylation and functional activation of Brk. Together, these results demonstrate that c-Src and Brk do not merely substitute for one another in mediating STAT5b transcriptional activation. Rather, Brk can function independently of c-Src, or these two kinases can work together to enhance STAT5b activity.

While Brk is not expressed in normal mammary epithelial cells, it is expressed in 60% of breast tumors – suggesting that Brk expression is regulated at the transcriptional level in breast cancer cells [16]. Furthermore, the DNA sequence of Brk isolated from gastrointestinal epithelial cells and that of Brk isolated from breast tumor cells are identical, suggesting that activating mutations in Brk are not accountable for Brk activity in breast cancer [18]. Located within the minimal functional promoter of Brk are NF-κB, Sp1, and STAT consensus binding sites, all known to play a role in tumorigenesis. Only NF-κB and Sp1, however, have thus far been shown to bind the Brk promoter [37]. Reports in human breast cancer samples have varied, showing that Brk correlates with HER2 and HER4 overexpression, as well as with estrogen receptor positivity [38-40]. There is also conflicting evidence showing a strong correlation between Brk staining by immunohistochemistry and tumor grade in one report [29], and another correlating Brk expression with long-term survival [39].

Our mRNA and protein analysis across a panel of human breast cancer cell lines showed no correlation of Brk expression with estrogen receptor status or EGFR/HER2 overexpression (Figure 4). We have, however, shown that knockdown of Brk significantly decreased DNA synthesis in the EGFR-overexpressing and c-Src-overexpressing breast cancer cell lines, SKBr3 and BT-20 (Figure 6). The effect of Brk knockdown on inhibiting basal DNA synthesis was comparable with that seen with STAT5b knockdown in both cell lines. Furthermore, simultaneous knockdown of both Brk and STAT5b had the same effect on decreasing DNA synthesis as their individual knockdowns, suggesting that this kinase and substrate converge upon a pathway ultimately leading to proliferation. Ostander and colleagues have shown that Brk knockdown decreases cell growth as well as epidermal growth factor-induced (or heregulin-induced) migration of breast cancer cells [29]. Additionally, knockdown of Brk decreases epidermal growth factor-induced (or heregulin-induced) cyclin D_1 _expression in breast cancer cells [29]. As STAT5b is known to regulate cyclin D_1 _through consensus sites in the promoter, this pathway may be one mechanism by which Brk and STAT5b together regulate increases in DNA synthesis. Given the evidence that we have provided for the functionally relevant regulation of STAT5b by Brk, further pursuing the role of this Brk–STAT5b pathway in breast cancer may provide important novel therapeutic targets.

## Conclusion

We have demonstrated that the nonreceptor tyrosine kinase Brk positively regulates STAT5b activity leading to proliferation of breast cancer cells. Based on our studies, we propose that HER2, EGFR, c-Src, and Brk all signal downstream to STAT5b to increase proliferation of breast cancer cells. We hypothesize that inhibition of STAT5b would obstruct the proliferative signal that is transmitted via these kinases resulting in decreased tumor growth.

## Abbreviations

BrdU = bromodeoxyuridine; Brk = breast tumor kinase; DMEM = Dulbecco's modified Eagle's medium; EGFR = epidermal growth factor receptor; FCS = fetal calf serum; NF = nuclear factor; PBS = phosphate-buffered saline; siRNA = small interfering RNA; STAT = signal transducer and activator of transcription.

## Competing interests

The authors declare that they have no competing interests.

## Authors' contributions

AMW performed all the experiments. Both AMW and CMS conceived the experiments, analyzed the data, and drafted the manuscript. Both authors read and approved the final manuscript.
